# PARP-1 Expression Influences Cancer Stem Cell Phenotype in Colorectal Cancer Depending on p53

**DOI:** 10.3390/ijms24054787

**Published:** 2023-03-01

**Authors:** Jose D. Puentes-Pardo, Sara Moreno-SanJuan, Jorge Casado, Julia Escudero-Feliu, David López-Pérez, Paula Sánchez-Uceta, Paula González-Novoa, Julio Gálvez, Ángel Carazo, Josefa León

**Affiliations:** 1 Instituto de Investigación Biosanitaria de Granada, ibs.GRANADA, 18012 Granada, Spain; 2 Departamento de Farmacología, Facultad de Farmacia, Universidad de Granada, 18011 Granada, Spain; 3 Cytometry and Microscopy Research Service, Instituto de Investigación Biosanitaria de Granada, ibs.GRANADA, 18012 Granada, Spain; 4 Centro de Investigación Biomédica en Red-Enfermedades Hepáticas y Digestivas (CIBER-ehd), 18011 Granada, Spain; 5 Unidad de Gestión de Microbiología, Hospital Universitario San Cecilio de Granada, 18016 Granada, Spain; 6 Unidad de Gestión Clínica de Aparato Digestivo, Hospital Universitario San Cecilio de Granada, 18016 Granada, Spain

**Keywords:** colorectal cancer, PARP-1, cancer stem cells, p53

## Abstract

Poly(ADP-ribose) polymerase-1 (PARP-1) is a protein involved in multiple physiological processes. Elevated PARP-1 expression has been found in several tumours, being associated with stemness and tumorigenesis. In colorectal cancer (CRC), some controversy among studies has been described. In this study, we analysed the expression of PARP-1 and cancer stem cell (CSC) markers in CRC patients with different p53 status. In addition, we used an in vitro model to evaluate the influence of PARP-1 in CSC phenotype regarding p53. In CRC patients, PARP-1 expression correlated with the differentiation grade, but this association was only maintained for tumours harbouring wild-type p53. Additionally, in those tumours, PARP-1 and CSC markers were positively correlated. In mutated p53 tumours, no associations were found, but PARP-1 was an independent factor for survival. According to our in vitro model, PARP-1 regulates CSC phenotype depending on p53 status. PARP-1 overexpression in a wild type p53 context increases CSC markers and sphere forming ability. By contrast, those features were reduced in mutated p53 cells. These results could implicate that patients with elevated PARP-1 expression and wild type p53 could benefit from PARP-1 inhibition therapies, meanwhile it could have adverse effects for those carrying mutated p53 tumours.

## 1. Introduction

Worldwide, colorectal cancer (CRC) ranks third in tumour type frequency, but second in terms of mortality [1]. Despite the decrease in CRC mortality achieved over the last few years, it remains high, especially in advanced stage cases, as a result of relapse after treatment and resistance to therapy [2]. The latter two factors have been associated with the presence of cancer stem cells (CSCs), a small subset of cancer cells also considered as the root for cancer initiation and metastasis [3]. These cells are characterized by the presence of several surface markers such as CD44, CD133 and CD326, an elevated ALDH1 activity, the overexpression of multidrug resistance associated proteins, and overactivation of DNA repair pathways [4,5]. Since these cells play a key role in cancer biology, in the last few years a deep effort has been made to find new features of the CSCs or specific targets with the aim of achieving more suitable therapies.

Poly(ADP-ribose) polymerase-1 (PARP-1) is the most known and abundant member of the PARP enzyme family consisting of 17 ADP-ribosylating enzymes. PARP-1 is mainly involved in the detection and repair of DNA damage through a process in which PARP-1 catalyses the polymerization of ADP-ribose monomers, PARylation or poly(ADP-ribosyl)ation, on target proteins [6]. In addition, PARP-1 is also involved in other molecular and cellular processes, including transcriptional regulation, DNA remodelling, hypoxic response, epithelial mesenchymal transition, angiogenesis, autophagy, and inflammation [7].

PARP-1 is overexpressed in several human cancers, including CRC [8,9,10,11], in which the expression of PARP-1 seems not to be homogenous in tumour cells, but it is highly expressed in CSCs compared with non-CSCs [12], indicating that PARP-1 could regulate CSC programming, as previously reported [7]. However, its relevance or function remains unknown. The fact that PARP-1 is overexpressed in tumour cells and contributes to vital processes for them suggest the idea of the use of PARP-1 inhibitors (PARPi) to fight cancer. In fact, olaparib, a PARPi approved by the FDA, is clinically used for the treatment of BRCA-mutated breast and ovarian cancers [13]. However, no clear effect has been found in clinical trials in which PARP-1 inhibitors have been used in monotherapy regimen in CRC [14]. Recently, a study revealed that PARP-1 seems to protect against the carcinogenic process but once it is initiated, PARP-1 expression facilitates tumour progression [11,15], indicating that this protein acts as a double-edged sword in CRC, suppressing tumour initiation but promoting inflammation-driven tumour progression [15]. Therefore, more research is needed to determine the parameters that influence the implication of PARP-1 in tumour development to potentially know the response to the treatment with PARPi in CRC.

The function of PARP-1 in a tumoral context is complex since there is a crosstalk between it and the dynamic of tumour microenvironment [7]. p53, a key protein involved in the response to oncogenic stress that is mutated in a large number of human cancers, seems to interact with PARP-1, although the potential effects are still unclear [16]. PARP- 1 does not seem to regulate p53 transactivation functions itself, but modulates them by promoting p53 nuclear localization [17]. Interestingly, p53 poly(ADP-ribosyl)ation occurs independently of p53 status, wild-type or mutated, as long as the mutations do not arise in specific regions at the C-terminus [17,18]. Recently, it has been described that parylated p53 might affect protein–protein interaction, and therefore p53 activity [18]. This interaction is bi-directional, and PARP-1 forms a complex with p53, but those with mutant p53 result in PARP-1 sequestered in the cytoplasm [19]. However, this arrest is determined by mutant p53 levels, being associated with chromatin and increased poly-ADP-ribosylated proteins in the nucleus when mutant p53 expression is high, but redistributed to the cytosol when its expression is low [20,21]. This PARP-1/p53 interaction is complex and its effects remain elusive.

Given the potential relationship between PARP-1 and p53, the aim of this study is to analyse the expression of PARP-1 in colorectal tumours with different p53 status in order to evaluate its influence on the CSC phenotype.

## 2. Results

### 2.1. PARP-1 Correlates with Clinical Outcomes in CRC Patients in a p53 Dependent Manner

**Figure 1:**
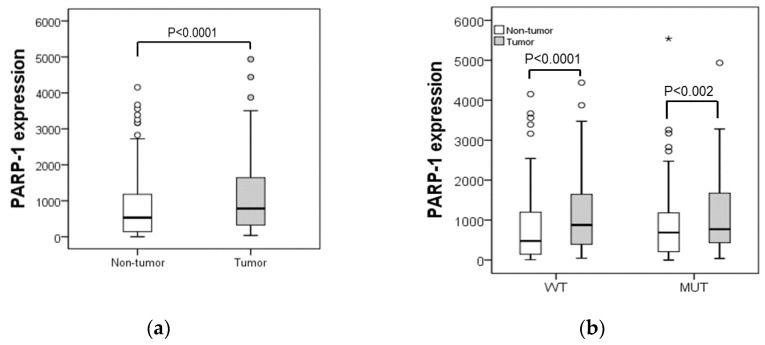
Box-plots representing PARP-1 expression in tumour and non-tumoral samples (**a**) from all patients and (**b**) patients stratified by p53 status in wild-type (WT) and mutated (MUT). Comparations were made by using non-parametric tests in two related samples. ° Represent outliers beyond 1.5 times the interquartile range (ICR); * Represent outliers beyond 3 times the ICR.

PARP-1 mRNA expression was measured in paired tumour and non-tumour tissues from CRC patients. Considering all cases analysed, PARP-1 was highly expressed in the tumoral tissue compared to their paired non-tumoral mucosa (*p* < 0.0001) (Figure 1a). Stratifying patients according to their p53 status, this tendency was maintained, the overexpression of PARP-1 in the tumoral tissue being significantly increased compared to their corresponding non-tumoral tissue in cases with p53 wild-type (*p* < 0.0001) and with p53 mutated (*p* < 0.002) (Figure 1b).

To examine the role of PARP-1 in CRC progression, the related clinicopathological parameters were investigated through statistical analysis (Table 1). The results showed that increased expression of PARP-1 was significantly associated with advanced dedifferentiation in the total of the tumours analysed (*p* = 0.042), and in tumours harbouring a wild-type p53 (*p* = 0.002). Nonetheless, no significant relationships were found between PARP-1 and age, gender, tumour location, pTNM stage, tumour stage, lymph node metastasis, and p53 mutations.

**Figure 2:**
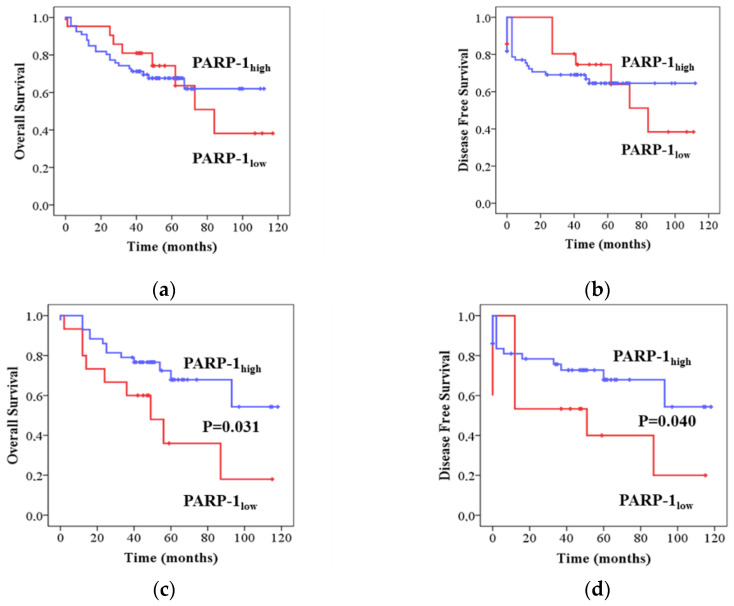
Kaplan–Meier curves depicting OS and DFS according to expression patterns of PARP-1 in (**a**,**b**) patients with p53 wild-type tumours and (**c**,**d**) patients with p53 mutated tumours. *p* values were calculated with the log-rank test.

To further evaluate the potential correlation between PARP-1 expression and patient outcome, we generated Kaplan–Meier survival curves from our cohort of patients by sorting out patients based on PARP-1 levels (high and low expression groups) and p53 status (wild-type or mutated). High PARP-1 expression was associated with significantly better OS (χ^2^ = 0.01, *p* = 0.031) and DFS (χ^2^ = 0.01, *p* = 0.040) in patients with mtp53 tumours (Figure 2c,d). OS and DFS were not significantly related to PARP-1 expression in wtp53 tumours (χ^2^ = 0.001, *p* = 0.972 and χ^2^ = 0.01, *p* = 0.998, respectively) (Figure 2a,b). Further, multivariate Cox regression for survival analysis showed PARP-1 expression as an independent prognostic factor for survival in CRC patients harbouring mutations in p53 (Table 2).

### 2.2. PARP-1 Regulates Tumourigenic Properties through the Regulation of Cancer Stem-Like Cells Phenotype

Since the PARP-1 mRNA level correlated with the differentiation grade, it was compared with CSC markers expression (Table 3). As can be seen, high expression of PARP-1 significantly correlated with high expression of CSC markers in the total and in p53 wild-type tumours. No significant correlation between these parameters was found in p53 mutated tumours.

**Figure 3:**
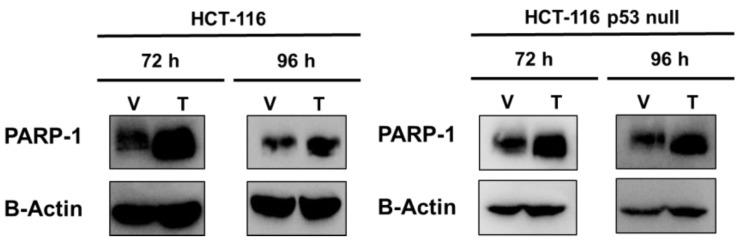
Cells were pCMV6-PARP1 or mock transfected, collected 72 and 96 h after transfection and analysed by Western blotting to ensure overexpression of PARP-1.

To further decipher the role of PARP-1 in CRC, we transiently overexpressed PARP- 1 in two human colorectal cancer cell lines with different status of p53: HCT-116 (p53 wild-type) and HCT-116 p53 null (p53 −/−) (Figure 3). We then compared the behaviour of the control cell lines (expressing only vector, V) with that of PARP-1-overexpressed isogenic daughter (pCMV6-PARP1) cells.

**Figure 4:**
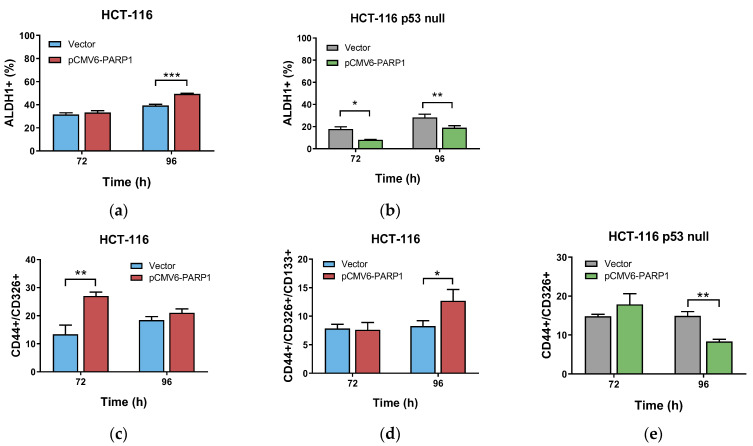
Cells were pCMV6-PARP1 or mock transfected (Vector), collected at 72 and 96 h after transfection and used to characterize the percentage of ALDH1+ cells by flow cytometry in total population (TP) of (**a**) HCT-116, (**b**) HCT-116 p53 null. In other experiments, cells were also collected at 72 and 96 h after pCMV6-PARP1 or empty vector transfections and used to quantify in TP (**c**) the percentage of CD144+/CD326+/CD133+ and (**d**) the percentage of CD44+/CD326+ cells in HCT-116, and (**e**) the percentage of CD44+/CD326+ cells in HCT-116 p53 null cells. Data represent the mean ± SD of two experiments performed in duplicate. * *p* < 0.05; ** *p* < 0.01; *** *p* < 0.001 vs. vector.

PARP-1 overexpression increased ALDH1+ activity in HCT-116 at 96 h after transfection and decreased it in HCT-116 p53 null cells at 72 and 96 h (Figure 4a,b). In HCT-116, transfection with pCMV6-PARP1 induced higher percentage of CD44_high_/CD326_high_ cells versus mock-transfected cells at 72 h although this difference was not significant at 96 h. On the contrary, we found a similar percentage of CD44_high_/CD326_high_/CD133_high_ cells at 72 h after transfection with pCMV6-PARP1 versus mock-transfected cells, and an increase in this percentage at 96 h. As previously reported, we did not find triple-labelled subpopulation cells in the HCT-116 p53 null cells [22]. In these cells, PARP-1 overexpression induced no changes in the percentage of CD44_high_/CD326_high_ cells versus mock-transfected cells at 72 h, whereas a decrease was found at 96 h after transfection. Altogether, these results indicate a phenotypic change induced due to PARP-1 overexpression in both HCT-116 and HCT-116 p53 null cells.

**Figure 5:**
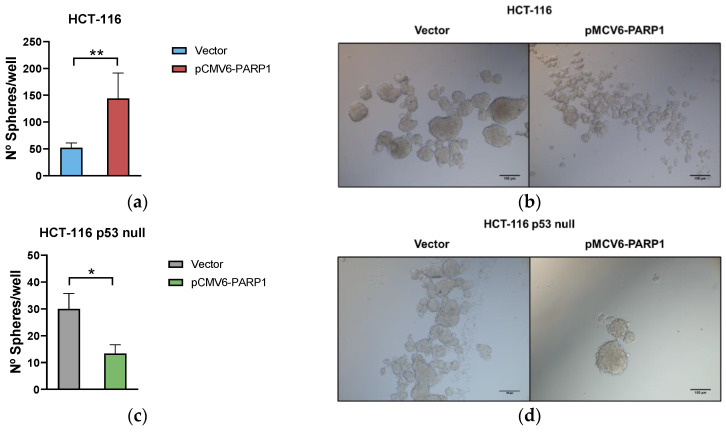
Influence of PARP-1 overexpression on sphere-forming ability of CRC in vitro. Number of spheres formed by cells obtained from mock and pCMV6-PARP1 (**a**) HCT-116 and (**b**) HCT-116 p53 null transiently transfected cells. Representative images of tumorospheres formed from pCMV6-PARP1, (**c**) HCT-116 and (**d**) HCT-116 p53 null transiently transfected cells. * *p* < 0.05; ** *p* < 0.01.

To further study whether such phenotypic changes affect the stemness of the cells, we analysed the self-renewal ability through the sphere formation assay after PARP-1 overexpression in HCT-116 and HCT-116 p53 null cells. As shown in Figure 5, after transfection with pCMV6-PARP1 the sphere number was higher (Figure 5a), although its size decreased in HCT-116 (Figure 5b). However, HCT-116 p53 null cells showed a minor sphere formation ability (Figure 5c) with similar size (Figure 5d) after PARP-1 overexpression.

## 3. Discussion

The contribution of PARP-1 in cancer initiation and progression is not totally understood despite it being overexpressed in several human cancer types [8,9,10]. Regarding CRC, little is known about the importance of PARP-1 expression in this tumour, beyond its overexpression in regard to the normal mucosa and being correlated with disease progression [11,15,23]. However, PARP-1 seems not to be the cause of carcinogenesis, since it may act as a tumour suppressor, likely due to its role in DNA repair. However, once a tumour occurs, PARP-1 facilitates its progression [15].

Similar to other studies, in this work we found PARP-1 was overexpressed in CRC samples compared to their healthy mucosa, however, in our cohort, the expression of PARP-1 was correlated to differentiation grade, specifically with dedifferentiated tumours, and not with tumour progression. Additionally, in our work, patients were stratified according to their p53 status, into wild-type p53 or mutated p53, which is not usually performed despite it being mutated in around 43% of cases, which may affect multiple pathways involved in tumour development [24]. In fact, once patients were stratified, the association between PARP-1 and the differentiation grade was only found in the wild-type p53 group, revealing PARP-1 may have different effects on clinicopathological characteristics depending on p53 context.

These data suggest that PARP-1 may also exert a different clinical outcome in terms of survival in CRC. In several solid tumours, including breast, ovarian, lung, liver, brain, oesophagus, pancreas, skin, stomach, and acute myeloid leukaemia, high expression of PARP-1 has been related to poorer survival rates [25,26]. On the contrary, there are studies in pancreatic and breast cancer that differ in how PARP-1 affects survival, suggesting that high PARP-1 expression is associated with better survival [27,28]. This indicate that PARP-1 could have different roles depending on the type of cancer, or even that, PARP-1 function could be highly influenced by the heterogeneity context. As far as we know, our work is the first in linking PARP-1 expression with survival rates, OS and DFS, in CRC. According to our data, a high PARP-1 expression is related to better survival rates, but only in those patients with mutated p53 status.

The difference in terms of survival among studies might be also attributed to differences in tumour type, study design, ethnicity, or different genomic context. Regarding the latter, we took into consideration differences in p53, which in fact influenced our study depending on its state [25,26,27,28]. The differences between the wild-type p53 and mutated p53 regarding PARP-1 expression could be a direct consequence of the mutation on p53, since those tumours are presumed to be more prone to genetic instability, which triggers DNA repair mechanism [29]. As the mutations on TP53 are diverse, being multiple associated with gain-of-function, some of them lead to damped repair mechanisms, which ultimately may make cells dependent on PARP-1 repair mechanisms to survive [30]. In fact, mutated p53 has been associated with increase of poly-ADP-ribosylated proteins in the nucleus [21]. PARP-1 hyperactivation may result in ATP depletion, toxic levels of poly-ADP-ribose, and mitochondria dysfunction, which cause a new programmed death mode called parthanatos [31]. Although this mechanism could be responsible for the differences observed in survival, this hypothesis requires more studies, since the knowledge about parthanatos is still naïve, and how the different p53 isoforms influence the different oncogenic gain-of-function still represents a black box.

Our data suggest that those differences could be due to the relationship of PARP-1 with the differentiation grade depending on p53. In wild-type p53 tumours, PARP-1 was associated with dedifferentiated tumour, and given the link between dedifferentiated tumours and the presence of CSCs cells [32], we next associated PARP-1 levels with different CSC marker levels. In our cohort of study, a high expression of PARP-1 is correlated with a high expression of CSC markers. In this sense similar results have been described in other studies, pointing out that CSCs express higher levels of PARP-1 than non-CSCs [33,34,35].

Once patients were stratified according to their p53 status, we found that in those patients with wild-type 53, a high PARP-1 expression was related to higher expression of CD133 and CD44 markers. The lack of association in the mutated p53 groups suggests that the preferential PARP-1 overexpression in CSCs may not be a direct consequence of the tumoral process per se, but a feature of the stem phenotype, as this type of cells possesses a DNA repair system which is very robust in order to maintain genomic integrity [36,37]. This has opened up the idea of the use of PARPi to target CSCs, but they failed to accurately treat cancer as a single therapy approach [14,33], and are required in combination with other chemotherapeutic agents [12]. However, the benefits of PARPi deteriorate due to the resistance appearance [38]. Additionally, it is important to note that the adverse effect of PARP-1 is in part assumed by its high baseline expression in CSCs, but most studies do not study how PARP-1 expression contributes or influences tumour progression during the disease [33].

In fact, our data suggest that PARP-1 only exerts detrimental activities in patients harbouring tumours with wtp53, but a high PARP-1 expression in mutated p53 tumours seems to be beneficial as it is an independent prognostic factor for survival, and these differences may be a result of a differential regulation of CSCs by PARP-1 depending on p53. To prove it, we developed an in vitro model with two cell lines with different p53 status and overexpressed PARP-1 transiently.

In this model, we found that the overexpression of PARP-1 increased the percentage of ALDH1+ population in HCT-116 cell line, meanwhile it was not altered or reduced in the HCT-116 p53 null line. This data was in concordance with the patient data. Since CSCs actually comprise a different subpopulations of stem-like cells [39], we also analysed by flow cytometry the percentage of cells expression in the CSC markers we used in the patient analysis. Similar results were found, and PARP-1 produces an increase of CD44_high_CD326_high_ and CD44_high_CD326_high_CD133_high_ population in the wild-type p53 cell line, and no changes or a decrease in the HCT-116 p53 null one. Additionally, the ability to form spheres was increased upon PARP-1 overexpression in the cells harbouring a wild type p53, meanwhile the lack of p53 resulted in a decrease of spheres when PARP-1 was overexpressed. These in vitro results confirmed the ones obtained from CRC patients and suggest that PARP-1 may exert benign or detrimental effects depending on the p53 status, conceivably via differential regulation of CSC phenotype in CRC.

This data may be cancer-type specific, since this study is the first one to shed light on the potential dual effect of PARP-1 in CRC, and the previous PARP-1 works have been mainly focused on the fact of PARP-1 being overexpressed in CSCs, discussed previously, and in the combination of PARP-1 inhibitors with other therapies [12,40]. Nonetheless, results supporting our data have been described in several cancers but have not been put in the context of p53, leading to controversy among some studies. A study using a pancreatic mouse cell line found that the inhibition of PARP-1 reduces CSC features, similar to the results found in neuroblastoma in which PARP-1 promotes stemness [41,42]. Both studies used cell lines harbouring a wild-type form of p53. On the other hand, a recent study in ovarian cancer showed that PARP inhibition reduces tumour size, although it enriches the CSC population within the tumour [43]. This result initiates controversy among studies and may lead to the idea that enrichment may be characteristic of the subpopulation studied or it is an implication of the research design, however, the latter study uses cell lines with mutated p53. This controversy may not be a controversy itself, but a consequence of the differential CSC regulation depending on p53 described in our study. This apparent relationship between PARP-1 and p53 would be supported by the fact that in CRC, patients with wild-type p53 may respond better to PARP inhibition than those with mutated p53 [44].

All these results are in line with our finding, indicating that PARP-1 regulates differentially stemness depending on p53 status, with the subsequent impact on patient prognosis. However, more research is required to confirm our results, and to study in depth the pathways that could induce this differential regulation.

## 4. Materials and Methods

### 4.1. Patients

Tumour samples and normal mucosa from 201 patients with primary sporadic CRC were prospectively provided by the Andalusian Tumour Bank Network (RBTA). The Ethics Committee of San Cecilio University Hospital approved the study (project code: PI-067/2013; date of approval: 24 January 2014), and all patients gave written informed consent for the post-surgery storage of samples and their use in biomedical research. The exclusion criteria were as follow: (1) Patients aged 18 or less and patients over 85; (2) patients receiving neoadjuvant chemotherapy; (3) patients diagnosed of hereditary CRC; (4) patients with a history of any other cancer.

Clinicopathological data of patients were also provided by the RBTA, including age, sex, tumour site, cell differentiation, clinical TNM stage, and lymph node metastasis. Patients were eligible for data collection if they were histologically diagnosed with CRC and previously treated with primary surgery (Table S1). The survival of the cohort was followed for 120 months.

### 4.2. Analysis of p53 Mutations in CRC Samples

Genomic DNA was extracted from tissue samples using QIAamp DNA Mini Kit (Qiagen, Hilden, Germany) following the commercial datasheet. The DNA was quantified using a NanoDrop ND-1000 (Implen GmbH, Munich, Germany) and its integrity was assessed by electrophoresis in agarose gel.

P53 mutations were determined as previously reported [45]. Briefly, mutations of TP53 in exons 2–10 of the tissue samples were analysed by PCR using specific primers (Table S2). PCR products were purified using Wizard SV gel and PCR clean-up system (Promega, Madison, WI, USA). Automated sequencing of the PCR products was performed using the 3130 XL Applied Biosystems, Foster City, CA, USA). The results were analysed using the software Chromas Lite 2.1.1 (St South Brisbane, QLD, Australia).

### 4.3. RNA Extraction and First-Strand cDNA Synthesis

Total RNA from tissue samples was isolated using the TRIzol reagent (Invitrogen, Life Technologies, Carlsbad, CA, USA). The amount of total RNA was determined by UV spectrophotometry, and RNA integrity was assessed by agarose gel electrophoresis. First-strand cDNA was prepared by reverse transcription with oligo-dT primers using a commercial cDNA synthesis kit (qScript™ cDNA Synthesis kit, Quanta Biosciences, Gaithersburg, MD, USA).

### 4.4. Real-Time PCR (RT-PCR)

Once retrotranscription was completed, 5 μL of the cDNA was amplified for 40 cycles, employing PerfeCTa SYBR Green SuperMix Kit (Quantabio, Beverly, MA, USA), and using specific primers for: PARP-1, CD44 and CD133, UBC, TBP, and RPS13 (Table S3). UBC, TBP, and RPS13 were used to normalize mRNA levels. For each target, a standard curve representing Ct values versus log cDNA dilution was produced. Additionally, PCR products were verified by melting profile and agarose gel electrophoresis to rule out nonspecific products and primer dimers.

### 4.5. Cell Culture

Two CRC cell lines with different p53 status were used: HCT-116 (p53 wild type) and HCT-116 p53 null (p53 −/−) obtained from Horizon Discovery (Cambridge- UK). Both cell lines were growing using RPMI 1640 medium (Gibco, Carlsbad, CA, USA) supplemented with 2 mM L-glutamine, and completed with 10% FBS and 1% antibiotic–antimycotic cocktail containing penicillin (100 U/mL), streptomycin (100 μg/mL), and amphotericin B (250 ng/mL) (Gibco, Carlsbad, CA, USA) under standard conditions (37 °C and 5% CO_2_ in a humidity atmosphere).

### 4.6. Transient Transfection

HCT-116 and HCT-116 p53 null cell lines were transfected with the plasmid pCMV6 containing the human PARP-1 gene and the corresponding empty vector (Origene Technologies, Rockville, MA, USA). For this purpose, 200.000 cells were seeded per well in a 6 well-plate. Once 60–70% confluence was reached, they were transfected with Lipofectamine 2000 (Thermo Fisher Scientific, Waltham, MA, USA) as a transfection reagent, with a lipofectamine/DNA ratio of 2.5, according to the manufacturer’s instructions.

### 4.7. Western Blotting

Proteins from whole cell homogenization were isolated using lysis buffer (RIPA buffer supplemented with phosphatase and protease inhibitors). After a 30 min incubation at 4 °C and the subsequent centrifugation at 16,000× *g* for 30 min, the amount of proteins was quantified through Bradford assay. Fifty micrograms of proteins was loaded into SDS-polyacrylamide gels and transferred to PVDF membrane using a Bio-Rad Trans-Blot Turbo Transfer System (Bio-Rad Laboratories, Inc., Hercules, CA, USA). The blots were probed with the appropriate antibodies for PARP-1 (Abcam, dilution 1:5000), ꞵ-Actin (Santa Cruz Biotechnology, Dallas, TX, USA, dilution 1:1000). As a secondary antibody, HRP-conjugated anti-rabbit or anti-mouse secondary antibody (Bio-Rad, dilution 1:50.000) was used. For detection, Amersham ECL Select Western Blotting Detection Reagent (GE Healthcare, Hatfield, UK) was applied before luminography).

### 4.8. ALDEFLUOR Assay

The aldefluor assay was performed following the manufacturer’s protocol (STEMCELL technologies, Vancouber, BC, Canada) with a few modifications. After treatments, cells were incubated with BODIPY-aminoacetaldehyde (BAAA), a fluorescent non-toxic substrate for ALDH, which was converted into BOD-IPY-aminoacate (BAA) and retained inside the cells. Viable ALDH1+ cells were quantified by flow cytometry on an FACS Aria III (BD Biosciences, San Jose, CA, USA). The specific inhibitor of ALDH, diethylam inobenzaldehyde (DEAB), was used to control for background fluorescence.

### 4.9. Phenotypic Characterization of CSCs

Cell surface marker levels of CSCs were determined with human anti-CD44-PE, anti-CD326-FITC, and anti-CD133-APC antibodies (Biolegend, San Diego, CA, USA). After 30 min of incubation at darkness and 4 °C, the samples were analysed using a BD FACSAria III flow cytometry (Becton Dickinson, BD Biosciences) at the Cytometry and Microscopy Research Service of the Biosanitary Research Institute of Granada (ibs.GRANADA).

### 4.10. Sphere Forming Assay

For self-renewal analysis, total population 3000 cells were resuspended in sphere culture medium (DMEM:F12, 1% penicillin/streptomycin, B27, 10 μg/mL ITS, 1 μg/mL hydrocortisone, 4 ng/mL heparin, 10 ng/mL EGF, 20 ng/mL FGF) in ultra-low attachment 24-well plates (Corning). Spheres greater than 25 μM in diameter were counted after 4 days by light microscopy at the Cytometry and Microscopy Research Service of the Biosanitary Research Institute of Granada (ibs.GRANADA).

### 4.11. Statistical Analysis

For each patient, mRNA levels of genes in tumour samples were normalized to mRNA levels in normal mucosa. Descriptive statistics was reported as medians with inter-quartile range (IQR) for continuous variables and as whole numbers and percentages for categorical variables. Low and high levels of genes were obtained through the median of the mRNA expression levels in our cohort of patients. Associations between clinicopathological features of CRC patients and gene expression were analysed with the Kruskal–Wallis and Mann–Whitney non-parametric tests. The Pearson’s test was used for the correlation analysis after transforming the variables applying natural logarithms. The Kaplan–Meier method was used to determine the cumulative probability of overall survival (OS) and disease-free survival (DFS), and the differences were evaluated using Log-rank tests. Prognostic factors were evaluated using univariate and multivariate analysis (Cox proportional hazards regression model). *p* values lower than 0.05 were considered significant. All confidence intervals (CIs) were stated at the 95% level. All statistical calculations were performed using SPSS software version 15.0 for Windows (IBM, Chicago, IL, USA).

## 5. Conclusions

PARP-1 is overexpressed in the tumour tissue compared to healthy mucosa, being associated with the differentiation grade. Surprisingly, this association is only maintained in tumours harbouring a wild type p53. In fact, in those patients with mutant p53, PARP- 1 is an independent prognostic factor for survival in CRC. The differences observed are due to the differential regulation of stemness exerted by PARP-1, promoting or not, depending on p53 status, wild-type or mutant, respectively. These results imply that PARP-1 could be used as a potential clinical tool for personalized medicine, since patients with elevated expression of PARP-1 and wild-type p53 could benefit from PARPi therapies, meanwhile it could be adverse for those carrying tumours with mutant p53 and high PARP-1 expression.

## Figures and Tables

**Table 1:** Relationship between PARP-1 expression and clinicopathological characteristics of patients at different status of *p53*.

		All ^1^		wtp53 ^2^		mtp53 ^3^	
Characteristic		Median ± CL	*p*	Median ± CL	*p*	Median ± CL	*p*
Age (y) *^,4^	<72	1.63 (0.94–2.73)	0.654	1.81 (1.10–3.03)	0.157	1.46 (0.84–2.46)	0.489
	≥72	1.52 (1.00–2.52)		1.44 (1.01–2.21)		1.60 (0.96–2.93)	
Gender *	Male	1.60 (0.98–2.89)	0.323	1.71 (1.09–2.93)	0.184	1.50 (0.88–3.20)	0.403
	Female	1.54 (0.96–2.37)		1.53 (0.97–1.96)		1.47 (0.92–2.50)	
Location *	Colon	1.62 (0.98–2.57)	0.430	1.71 (1.05–2.44)	0.325	1.51 (0.91–2.81)	0.540
	Rectum	1.24 (0.86–3.09)		1.62 (0.86–3.59)		1.62 (0.98–26.25)	
DG ^†&^	Well	1.16 (0.58–2.50)	0.042	1.10 (0.56–1.43)	0.002	1.23 (0.57–4.97)	0.980
	Moderately	1.77 (1.11–2.57)		1.78 (1.19–2.63)		1.65 (0.99–2.52)	
	Poor	1.54 (0.92–3.28)		2.32 (0.91–4.55)		1.27 (0.92–4.80)	
pTNM Stage *	Stage I + II	1.69 (1.07–2.66)	0.501	1.69 (1.10–2.65)	0.785	1.70 (0.92–2.74)	0.445
	Stage III + IV	1.58 (0.96–2.69)		1.57 (0.92–2.29)		1.54 (0.88–2.69)	
Tumour stage *	T1 + T2	1.78 (1.07–3.25)	0.505	1.61 (1.17–2.40)	0.732	2.01 (0.57–4.08)	0.942
	T3 + T4	1.57 (0.95–2.52)		1.56 (0.96–2.73)		1.50 (0.92–2.76)	
LNM *^,#^	Absent	1.42 (0.78–2.48)	0.503	1.58 (0.97–2.52)	0.850	1.46 (0.53–3.33)	0.383
	Present	1.50 (0.92–2.10)		1.58 (0.90–2.03)		1.27 (0.90–2.54)	

* Analysis was performed using non-parametric Mann–Whitney *U* test for independent samples or ^†^ Kruskal–Wallis test for independent samples; ^#^ Lymph Node Metastasis; ^&^ Differentiation Grade; ^1^ All cases studied; ^2^ p53 wild-type tumours; ^3^ p53 mutated tumours; ^4^ Dichotomized by the median.

**Table 2:** Cox regression of prognostic factors for CRC.

		wtp53 ^1^		mtp53 ^2^	
Variables		HR [95 % CI]	*p*	HR [95 % CI]	*p*
PARP-1 *	Low	1		1	
	High	1.30 [0.57, 3.00]	0.532	0.36 [0.15, 0.88]	0.025
**Age**	≤72	1		1	
	>72	2.27 [0.91, 5.64]	0.078	2.32 [0.93, 5.79]	0.072
**Gender**	Female	1		1	
	Male	1.54 [0.70, 3.38]	0.286	1.21 [0.45, 3.26]	0.709
**TNM**	Stage I + II	1		1	
	Stage III + IV	1.86 [0.83, 4.16]	0.130	1.72 [0.59, 5.04]	0.322
**C ^3^ and/or R ^4^**	No	1		1	
	Yes	0.66 [0.28, 1.56]	0.341	0.74 [0.25, 2.62]	0.602

* Categorized by the median; HR: hazard ratio; CI: confidence interval; ^1^ p53 wild-type tumours; ^2^ p53 mutated tumours; ^3^ chemotherapy; ^4^ radiotherapy.

**Table 3:** Relationship of PARP-1 and CSC marker expression in our cohort of CRC patients according to p53 status.

		All ^1^			wtp53 ^2^			mtp53 ^3^		
		PARP-1			PARP-1			PARP-1		
		Low	High	*p*	Low	High	*p*	Low	High	*p*
**CD133_low_CD44_low_**		30 (61.2)	19 (38.8)	<0.001 *	17 (53.1)	15 (46.9)	<0.001 *	13 (76.5)	4 (23.5)	0.144
**CD133_high_CD44_high_**		13 (22.4)	45 (77.6)		6 (14.0)	37 (86.0)		7 (46.7)	8 (53.3)	

* Differences were analysed using the Chi-squared test or the Fisher’s exact test; ^1^ All cases studied; ^2^ p53 wild-type tumours; ^3^ p53 mutated tumours.

## Data Availability

The data are contained within this article.

## Supplementary Materials

The following supporting information can be downloaded at: https://www.mdpi.com/article/10.3390/ijms24054787/s1.

## References

[1] Sung H., Ferlay J., Siegel R.L., Laversanne M., Soerjomataram I., Jemal A., Bray F. (2021). Global Cancer Statistics 2020: GLOBOCAN Estimates of Incidence and Mortality Worldwide for 36 Cancers in 185 Countries. CA Cancer J. Clin..

[2] Van Der Jeught K., Xu H.C., Li Y.J., Lu X.B., Ji G. (2018). Drug resistance and new therapies in colorectal cancer. World J. Gastroenterol..

[3] Das P.K., Islam F., Lam A.K. (2020). The Roles of Cancer Stem Cells and Therapy Resistance in Colorectal Carcinoma. Cells.

[4] Cho Y., Kim Y.K. (2020). Cancer Stem Cells as a Potential Target to Overcome Multidrug Resistance. Front. Oncol..

[5] Batlle E., Clevers H. (2017). Cancer stem cells revisited. Nat. Med..

[6] Ray Chaudhuri A., Nussenzweig A. (2017). The multifaceted roles of PARP1 in DNA repair and chromatin remodelling. Nat. Rev. Mol. Cell Biol..

[7] Martí J.M., Fernández-Cortés M., Serrano-Sáenz S., Zamudio-Martinez E., Delgado-Bellido D., Garcia-Diaz A., Oliver F.J. (2020). The Multifactorial Role of PARP-1 in Tumor Microenvironment. Cancers.

[8] Gonçalves A., Finetti P., Sabatier R., Gilabert M., Adelaide J., Borg J.P., Chaffanet M., Viens P., Birnbaum D., Bertucci F. (2011). Poly(ADP-ribose) polymerase-1 mRNA expression in human breast cancer: A meta-analysis. Breast Cancer Res. Treat..

[9] Byers L.A., Wang J., Nilsson M.B., Fujimoto J., Saintigny P., Yordy J., Giri U., Peyton M., Fan Y.H., Diao L. (2012). Proteomic profiling identifies dysregulated pathways in small cell lung cancer and novel therapeutic targets including PARP1. Cancer Discov..

[10] Ossovskaya V., Koo I.C., Kaldjian E.P., Alvares C., Sherman B.M. (2010). Upregulation of poly (ADP-Ribose) polymerase-1 (PARP1) in triple-negative breast cancer and other primary human tumor types. Genes Cancer.

[11] Nosho K., Yamamoto H., Mikami M., Taniguchi H., Takahashi T., Adachi Y., Imamura A., Imai K., Shinomura Y. (2006). Overexpression of poly(ADP-ribose) polymerase-1 (PARP-1) in the early stage of colorectal carcinogenesis. Eur. J. Cancer.

[12] Jarrar A., Lotti F., DeVecchio J., Ferrandon S., Gantt G., Mace A., Karagkounis G., Orloff M., Venere M., Hitomi M. (2019). Poly(ADP-Ribose) Polymerase Inhibition Sensitizes Colorectal Cancer-Initiating Cells to Chemotherapy. Stem Cells.

[13] Pilié P.G., Tang C., Mills G.B., Yap T.A. (2018). State-of-the-art strategies for targeting the DNA damage response in cancer. Nat. Rev. Clin. Oncol..

[14] Leichman L., Groshen S., O’Neil B.H., Messersmith W., Berlin J., Chan E., Leichman C.G., Cohen S.J., Cohen D., Lenz H.-J. (2016). Phase II Study of Olaparib (AZD-2281) After Standard Systemic Therapies for Disseminated Colorectal Cancer. Oncologist.

[15] Dörsam B., Seiwert N., Foersch S., Stroh S., Nagel G., Begaliew D., Diehl E., Kraus A., McKeague M., Minneker V. (2018). PARP-1 protects against colorectal tumor induction, but promotes inflammation-driven colorectal tumor progression. Proc. Natl. Acad. Sci. USA.

[16] Wiman K.G. (2013). p53 talks to PARP: The increasing complexity of p53-induced cell death. Cell Death Differ..

[17] Kanai M., Hanashiro K., Kim S.H., Hanai S., Boulares A.H., Miwa M., Fukasawa K. (2007). Inhibition of Crm1–p53 interaction and nuclear export of p53 by poly(ADP-ribosyl)ation. Nat. Cell Biol..

[18] Fischbach A., Krüger A., Hampp S., Assmann G., Rank L., Hufnagel M., Stöckl M.T., Fischer J.M.F., Veith S., Rossatti P. (2018). The C-terminal domain of p53 orchestrates the interplay between non-covalent and covalent poly(ADP-ribosyl)ation of p53 by PARP1. Nucleic Acids Res..

[19] Wesierska-Gadek J., Schmid G. (2000). Overexpressed poly(ADP-ribose) polymerase delays the release of rat cells from p53-mediated G(1) checkpoint. J. Cell. Biochem..

[20] Qiu W.G., Polotskaia A., Xiao G., Di L., Zhao Y., Hu W., Philip J., Hendrickson R.C., Bargonetti J. (2017). Identification, validation, and targeting of the mutant p53-PARP-MCM chromatin axis in triple negative breast cancer. npj Breast Cancer.

[21] Polotskaia A., Xiao G., Reynoso K., Martin C., Qiu W.G., Hendrickson R.C., Bargonettia J. (2015). Proteome-wide analysis of mutant p53 targets in breast cancer identifies new levels of gain-of-function that influence PARP, PCNA, and MCM4. Proc. Natl. Acad. Sci. USA.

[22] Ríos-Arrabal S., Puentes-Pardo J.D., Moreno-Sanjuan S., Szuba Á., Casado J., García-Costela M., Escudero-Feliu J., Verbeni M., Cano C., González-Puga C. (2021). Endothelin-1 as a mediator of heme oxygenase-1-induced stemness in colorectal cancer: Influence of p53. J. Pers. Med..

[23] Dziaman T., Ludwiczak H., Ciesla J.M., Banaszkiewicz Z., Winczura A., Chmielarczyk M., Wisniewska E., Marszalek A., Tudek B., Olinski R. (2014). PARP-1 Expression is Increased in Colon Adenoma and Carcinoma and Correlates with OGG1. PLoS ONE.

[24] Liebl M.C., Hofmann T.G. (2021). The role of p53 signaling in colorectal cancer. Cancers.

[25] Li X., Li C., Jin J., Wang J., Huang J., Ma Z., Huang X., He X., Zhou Y., Xu Y. (2018). High PARP-1 expression predicts poor survival in acute myeloid leukemia and PARP-1 inhibitor and SAHA-bendamustine hybrid inhibitor combination treatment synergistically enhances anti-tumor effects. eBioMedicine.

[26] Thakur N., Yim K., Abdul-Ghafar J., Seo K.J., Chong Y. (2021). High poly(ADP-ribose) polymerase expression does relate to poor survival in solid cancers: A systematic review and meta-analysis. Cancers.

[27] Klauschen F., von Winterfeld M., Stenzinger A., Sinn B.V., Budczies J., Kamphues C., Bahra M., Wittschieber D., Weichert W., Striefler J. (2012). High nuclear poly-(ADP-ribose)-polymerase expression is prognostic of improved survival in pancreatic cancer. Histopathology.

[28] Aiad H.A., Kandil M.A.H., El-Tahmody M.A., Abulkheir I.L., Abulkasem F.M., Elmansori A.A., Aleskandarany M.A. (2015). The prognostic and predictive significance of PARP-1 in locally advanced breast cancer of Egyptian patients receiving neoadjuvant chemotherapy. Appl. Immunohistochem. Mol. Morphol..

[29] Mantovani F., Collavin L., Del Sal G. (2018). Mutant p53 as a guardian of the cancer cell. Cell Death Differ..

[30] Liu D.P., Song H., Xu Y. (2010). A common gain of function of p53 cancer mutants in inducing genetic instability. Oncogene.

[31] Huang P., Chen G., Jin W., Mao K., Wan H., He Y. (2022). Molecular Mechanisms of Parthanatos and Its Role in Diverse Diseases. Int. J. Mol. Sci..

[32] Friedmann-Morvinski D., Verma I.M. (2014). Dedifferentiation and reprogramming: Origins of cancer stem cells. EMBO Rep..

[33] Gilabert M., Launay S., Ginestier C., Bertucci F., Audebert S., Pophillat M., Toiron Y., Baudelet E., Finetti P., Noguchi T. (2014). Poly(ADP-Ribose) Polymerase 1 (PARP1) Overexpression in Human Breast Cancer Stem Cells and Resistance to Olaparib. PLoS ONE.

[34] Valencia-González H.A., Ruíz G., Ortiz-Sánchez E., García-Carrancá A. (2019). Cancer stem cells from tumor cell lines activate the DNA damage response pathway after ionizing radiation more efficiently than noncancer stem cells. Stem Cells Int..

[35] Venere M., Hamerlik P., Wu Q., Rasmussen R.D., Song L.A., Vasanji A., Tenley N., Flavahan W.A., Hjelmeland A.B., Bartek J. (2014). Therapeutic targeting of constitutive PARP activation compromises stem cell phenotype and survival of glioblastoma-initiating cells. Cell Death Differ..

[36] Vitale I., Manic G., De Maria R., Kroemer G., Galluzzi L. (2017). DNA Damage in Stem Cells. Mol. Cell.

[37] Zhao B., Zhang W.D., Duan Y.L., Lu Y.Q., Cun Y.X., Li C.H., Guo K., Nie W.H., Li L., Zhang R. (2015). Filia Is an ESC-Specific Regulator of DNA Damage Response and Safeguards Genomic Stability. Cell Stem Cell.

[38] Li H., Liu Z.Y., Wu N., Chen Y.C., Cheng Q., Wang J. (2020). PARP inhibitor resistance: The underlying mechanisms and clinical implications. Mol. Cancer.

[39] Tang D.G. (2012). Understanding cancer stem cell heterogeneity and plasticity. Cell Res..

[40] Qin C., Ji Z., Zhai E., Xu K., Zhang Y., Li Q., Jing H., Wang X., Song X. (2022). PARP inhibitor olaparib enhances the efficacy of radiotherapy on XRCC2-deficient colorectal cancer cells. Cell Death Dis..

[41] Quiñonero F., Mesas C., Muñoz-Gámez J.A., Jiménez-Luna C., Perazzoli G., Prados J., Melguizo C., Ortiz R. (2022). PARP1 inhibition by Olaparib reduces the lethality of pancreatic cancer cells and increases their sensitivity to Gemcitabine. Biomed. Pharmacother..

[42] Long W., Zhao W., Ning B., Huang J., Chu J., Li L., Ma Q., Xing C., Wang H.Y., Liu Q. (2018). PHF20 collaborates with PARP1 to promote stemness and aggressiveness of neuroblastoma cells through activation of SOX2 and OCT4. J. Mol. Cell Biol..

[43] Bellio C., DiGloria C., Foster R., James K., Konstantinopoulos P.A., Growdon W.B., Rueda B.R. (2019). PARP inhibition induces enrichment of DNA repair-proficient CD133 and CD117 positive ovarian cancer stem cells. Mol. Cancer Res..

[44] Smeby J., Kryeziu K., Berg K.C.G., Eilertsen I.A., Eide P.W., Johannessen B., Guren M.G., Nesbakken A., Bruun J., Lothe R.A. (2020). Molecular correlates of sensitivity to PARP inhibition beyond homologous recombination deficiency in pre-clinical models of colorectal cancer point to wild-type TP53 activity. eBioMedicine.

[45] Casado J., Iñigo-Chaves A., Jiménez-Ruiz S.M., Ríos-Arrabal S., Carazo-Gallego Á., González-Puga C., Núñez M.I., Ruíz-Extremera Á., Salmerón J., León J. (2017). AA-NAT, MT1 and MT2 Correlates with Cancer Stem-Like Cell Markers in Colorectal Cancer: Study of the Influence of Stage and p53 Status of Tumors. Int. J. Mol. Sci..

