# Supplementary material for: PARP-1 Expression Influences Cancer Stem Cell Phenotype in Colorectal Cancer Depending on p53
Source: Int J Mol Sci. 2023 Mar 1;24(5):4787. doi: 10.3390/ijms24054787 (PMC10002521; doi:10.3390/ijms24054787)
Supplement: Supplementary file 1 [file ijms-24-04787-s001.zip › ijms-2194462-supplementary.pdf]

Supplementary Table S1. Characteristics of the patients include in the study

Characteristics	N (%)
**Gender**	
Female	125 (62.4)
Male	76 (36.6)
**Location**	
Colon	189 (94)
Rectum	12 (6)
**Histological Type**	
Well differentiated	42 (21.0)
Moderately differentiated	136 (67.7)
Poorly differentiated	23 (11.3)
**pTMN Stage**	
Stage I	22 (11.1)
Stage II	77 (38.1)
Stage III	83 (41.3)
Stage IV	19 (9.5)
**Nº of lymph nodes removed**	
<12	71 (35.5)
≥12	130 (64.5)

Supplementary Table S2. Primers used to analyze P53 mutations

TP53	Forward	Reverse
Exon 2-4	agctgtctcagacactggcatggtgttgg	cactgacaggaagccaaagggtgaagagg
Exon 5-6	gttgctttatctgttcacttgtgccctgac	tagggaggtcaatatagcagcaggagaaag
Exon 7-9	cagcctgggcgacagagcgagattccatc	aaccaggagccattgtctttgaggcatcac
Exon 10	tacttgaagtgcagtttctactaaatgcatg	aggaagactaaaaaaatgtctgtgcagggc

Supplementary Table S3. Primers used to determine PARP-1, CD44, CD133, UBC, TBP and RPS13 expression

Gene	Forward	Reverse
PARP-1	agggcaagcacagtgtcaaa	tacccatcagcaacttagcg
CD44	gctttcaatagcaccttgcccacaatgg	aaagaggtcctgtcctgtccaaatcttcc
CD133	tccacagaaatttacctacattgg	cagcagagagcagatgacca
UBC	tgggatgcaaatcttcgtgaagaccctgac	accaagtgcagagtggactctttctggatg
TBP	ttgtccttttgccatttgctgggctcctc	gtcatcagtggagacggtttccatttaacc
RPS13	ggtgttgcacaagtacgttttgtgacaggc	tcatatttccaattgggagggaggactcgc
